# Protection and Pathology in *Leishmania braziliensis* Infection

**DOI:** 10.3390/pathogens11040466

**Published:** 2022-04-14

**Authors:** Augusto M. Carvalho, Olívia Bacellar, Edgar M. Carvalho

**Affiliations:** 1 Laboratório de Pesquisas Clínicas (LAPEC), Instituto Gonçalo Moniz, FIOCRUZ, Salvador 40296710, Brazil; augustomarcelino1@hotmail.com; 2 Immunology Service, Professor Edgard Santos University Hospital, Federal University of Bahia, Salvador 40110160, Brazil; olivinhaufba@gmail.com; 3 National Institute of Science and Technology in Tropical Diseases (INCT-DT), CNPq, Salvador 40296710, Brazil

**Keywords:** leishmaniasis, *Leishmania braziliensis*, host parasite infection, protection, pathology, American tegumentary leishmaniasis (ATL)

## Abstract

*Leishmania* killing is mediated by IFN-γ-activated macrophages, but IFN-γ production and macrophage activation are insufficient to control *L. braziliensis* infection. In American tegumentary leishmaniasis (ATL), pathology results from an exaggerated inflammatory response. This report presents an overview of our contributions regarding ATL pathogenesis, highlighting future directions to improve the management of *L. braziliensis* infection. Monocytes and lymphocytes from individuals exposed to *L. braziliensis* but who do not develop CL, i.e., subclinical infection (SC), exhibit lower respiratory burst and IFN-γ production, yet more efficiently kill *L. braziliensis*. As vaccines aimed at inducing IL-12 and IFN-γ do not sufficiently prevent CL, the elucidation of how subjects with SC infection kill Leishmania may lead to new approaches to controlling ATL. While inflammation arising from the recruitment of inflammatory cells via chemokines induced by IFN-γ and TNF or IL-17 is observed and contributes to pathology, cytotoxic CD8^+^ T cells and NK cells play a key role in the pathogenesis of *L. braziliensis* infection. The increased transcription of genes related to inflammation and cytotoxicity, e.g., granzyme A, granzyme B, NLRP3 and IL-1β, has been documented in CL tissue samples. The release of products by killed cells leads to NLRP3 inflammasome activation, IL-1β production and additional damage to skin and mucosal tissues. The use of drugs that downmodulate the inflammatory response in combination with chemotherapy improves the ATL cure rate and decreases healing time.

## 1. Introduction

Studies investigating the immunology of leishmaniasis were performed in experimental models. As leishmania is an intracellular pathogen and considering that the control of parasite proliferation is mediated by the cellular immune response in such cases, emphasis was placed on the role of T cells in the pathogenesis of infection by *Leishmania* spp. The documentation that CD4^+^ T cells are a heterogeneous cell population consisting of two subsets, Th1 and Th2, has expanded our knowledge on the control and pathological aspect of infectious diseases. CD4^+^Th1 cells are the main cell type producing IFN-γ, while IL-4 is the major cytokine expressed by CD4^+^ Th2 cells. In leishmaniasis, while BALB/c mice are known to be susceptible to *L. major* infection, the C57BL/6 strain is able to control parasite multiplication and thus prevent the development of cutaneous lesions. In this context, the abrogation of IFN-γ expression in C57BL/6 mice was associated with parasite proliferation, while the blockade of IL-4 made BALB/c mice resistant to *L. major* infection [1,2]. The differentiation of Th0 to Th1 cells is mediated mainly by IL-12 produced by dendritic cells and macrophages; it was later shown that IL-10 plays a critical role in the downmodulation of the Th1 immune response in leishmaniasis, and consequently in parasite survival and proliferation [3]. In human cutaneous leishmaniasis (CL) caused by *Leishmania (Viannia) braziliensis*, a predominant Th1 immune response is observed, with few parasites documented under histopathologic analysis. Alternatively, the impairment of Th1 immune response in visceral leishmaniasis leads parasites to proliferate and disseminate to the spleen, liver, lymph nodes and bone marrow [4]. However, strong evidence suggests that the Th1 vs. Th2 paradigm alone does not fully explain the pathogenesis of human CL caused by *L. braziliensis*. Importantly, a Th2-type immune response is not evidenced in tegumentary leishmaniasis or even in diffuse cutaneous leishmaniasis (DCL), which is associated with an impairment Th1 immune response [5]. The strong Th1 immune response observed in *L. braziliensis*-infected subjects does not lead to parasite eradication in macrophages; the resulting parasite persistence contributes to an exaggerated inflammatory response with pathological consequences [6]. In this scenario, the control and pathology of *L. braziliensis* is determined by a complex interplay involving the participation of a variety of cell types and many cytokines, as well as other molecules. Recent evidence suggests that CD4^+^ and CD8^+^ T cells, NK cells, macrophages and, to a lesser extent, neutrophils and B cells, may all participate in both the control and pathology of *L. braziliensis* infection [7,8,9]. In this overview, we summarize data from epidemiological, clinical and immunological studies performed in the municipality of Corte de Pedra, Bahia-Brazil, one of the most important areas of *L. braziliensis* transmission in Latin America. Additionally, we perform a critical analysis of the current gaps in our understanding of the pathogenesis of American tegumentary leishmaniasis (ATL) and propose potential courses of investigation for further study aimed at controlling parasite survival and attenuating disease pathology in an attempt to improve the management of *L. braziliensis* infection.

## 2. Results

### 2.1. Immune Response in the Early Phase of L. braziliensis Infection

CL is characterized by a well-delimited, round-shaped ulcer with raised borders; however, this feature is only observed 2–4 weeks after the dermal inoculation of *L. braziliensis*. Upon the establishment of *L. braziliensis* infection, the first observed manifestation is a small nonulcerated papular lesion, associated with exuberant satellite lymphadenopathy, the phase of disease known as early cutaneous leishmaniasis (E-CL) [10]. In E-CL, small amounts of Th1 cytokines IFN-γ and TNF are produced. However, the low-grade Th1 immune response becomes quickly reversed and 2 to 4 weeks later, enhanced levels of IFN-γ and TNF are observed together with the expression of cytokines CXCL-9, CXCL-10, IL-1β and IL-6 [11]. The association between an enhanced inflammatory response and ulcer development during the evolution from E-CL to CL was detected via initial evidence demonstrating the participation of the host immune response in the pathology of *L. braziliensis* infection. Of note, while in most infectious diseases early treatment is associated with an improved and rapid response to therapy, in E-CL antimony therapy produced a cure rate of 27%, compared to more than 60% in patients with ulcerated classic CL [12].

As expected, a large portion of individuals living in the endemic area under study have been exposed to *L. braziliensis* infection. The majority of these individuals do not develop CL and are recognized as having subclinical (SC) *L. braziliensis* infection. SC *L. braziliensis*-infection is characterized by a positive delayed-type hypersensitivity (DTH) test employing soluble leishmania antigen (SLA) and/or the production of IFN-γ in the supernatant of whole blood cells stimulated with SLA [13,14].

### 2.2. Immunological Response in Different Clinical Forms of L. braziliensis Infection

**Figure 1:**
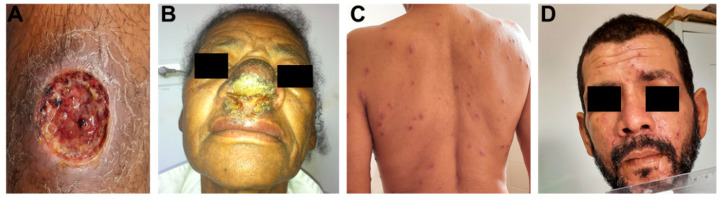
Clinical presentation of ATL caused by *Leishmania braziliensis*. (**A**) Male patient with CL presenting ulcerated lesion on the right leg. (**B**) Female patient with ML, displaying large ulcer on nasal mucosal. (**C**,**D**) Male patient with DL presenting mixed lesions (acneiform, crusted papules, superficial nodules and ulcers) on the trunk (**C**) and face (**D**).

CL is the most common clinical form of *L. braziliensis* infection, observed in around 90% of subjects who develop ATL in the endemic area around Corte de Pedra. Figure 1 shows the main clinical forms of ATL caused by *L. braziliensis*. Approximately 3% of CL patients develop mucosal leishmaniasis (ML), 6% present disseminated leishmaniasis (DL) and atypical CL is documented in around 1% of cases. ML, or mucocutaneous leishmaniasis, is a severe form of *L. braziliensis* resulting in the destruction of facial structures [15]. Notably, this severe presentation results in part from delayed disease diagnosis.

In the past, ML was mainly diagnosed in patients who developed CL months or even years prior to presenting mucosal disease. The absence of a typical cutaneous ulcer and the limited access to medical assistance by poor rural populations restricted ML diagnosis to more advanced phases of disease involving nasal septum rupture. However, recent findings indicate higher frequencies (up to 30%) of patients concomitantly presenting cutaneous and mucosal disease, and it has become clear that pathology in the nasal mucosa is highly variable [15,16]. Initially, patients presented nodular lesions and later developed superficial ulcers followed by deep ulcers, nasal septal perforation and damage to other facial tissues, including pharyngeal and laryngeal involvement [15]. A more exacerbated inflammatory response is seen in ML versus CL, yet parasites are scarcely found in tissue despite more evident tissue damage compared to CL. In addition to producing more IFN-γ and TNF, high levels of IL-17 are seen in ML, which is more highly expressed in mucosal tissue than in CL lesions [17]. Moreover, as more CD8^+^ T cells expressing granzyme A are documented in ML than in CL tissue, both IL-17 and cytotoxic CD8^+^ T cells participate in the pathogenesis of ML [18].

Disseminated leishmaniasis (DL) is characterized by at least 10 and up to more than 1000 acneiform, nodular and ulcerated lesions across at least two parts of the body [19]. The primary lesion in DL is an ulcer, similar to that observed in CL, and parasite dissemination occurs some weeks after infection, when patients abruptly present fever and chills lasting a day followed by the appearance of multiple lesions [19]. ML is observed in about 40% of patients with DL, with most patients presenting only nodules or superficial ulcers in the nasal cavity, indicating a brief duration of illness in the nasal mucosa [19]. Our investigations in the endemic area have shown that *L. braziliensis* is polymorphic and isolates causing DL present genotypic differences compared to those that cause CL [20]. These genotypic differences among isolates have also been associated with variable clinical forms of disease and therapeutic failure [20,21,22]. The parasites causing DL become more internalized and proliferate to a greater extent in macrophages compared to isolates from CL [23]. However, host factors also participate in the pathogenesis of DL, as monocytes taken from DL patients were found to be more permissive to proliferation by DL isolates than CL monocytes. Evidence suggests the role of B cells in the pathogenesis of ATL, as antibodies are produced in all clinical forms of *L. braziliensis* infection, and DL patients produce more antibodies than those with CL [24]. B cells and plasma cells are more frequent in tissue biopsied from DL patients compared to CL, and high levels of IgG anti-*L. braziliensis* antibodies were found to be associated with disease severity [24].

Few CL patients from the endemic area of Corte de Pedra present atypical cutaneous lupoid, sporotrichosis, vegetative or verrucous lesions, or lesions with multiple nodules. These atypical lesions may occur in patients with an impaired T cell response, such as pregnant women or HIV-co-infected individuals; however, in the majority of atypical CL cases, subjects are otherwise healthy, are not using immunosuppressive drugs and do not present comorbidities [25]. The isolates of *L. braziliensis* causing atypical CL exhibit genotypic difference from those associated with classic CL [21]. Table 1 details the main differences in immunological response in patients with diverse clinical forms of *L. braziliensis* infection.

*L. braziliensis* infection is characterized by an inflammatory reaction mediated by both innate and adaptative immune responses. *L. braziliensis*-infected patients develop a predominantly type 1 immune response with elevated production of pro-inflammatory cytokines. This exaggerated production of pro-inflammatory cytokines is observed in CL, atypical CL and ML. However, while higher expressions of IFN-γ and TNF is typical in CL and ML patients, peripheral blood mononuclear cells (PBMC) from patients with atypical CL and ML produce more IL-17 than cells from other clinical forms of *L. braziliensis* infection. IL-17 is downmodulated by IFN-γ and high production of IFN-γ occurs in *L. braziliensis* infection, which may prevent substantially enhanced IL-17 synthesis during the course of *Leishmania* infection. However, the higher IL-17 expression noted in cells from ML patients compared to CL has been associated with neutrophil recruitment and tissue damage in ML [17,26]. IL-17 is also produced at high levels in patients with atypical forms of CL [21]. In DL and in subjects with SC *L. braziliensis* infection, a lower expression of TNF and IFN-γ occurs compared to CL [14,23]. However, a predominant Th1 immune response is also observed in DL, as well as in subjects with SC infection, due to elevated IFN-γ levels compared to IL-4.

### 2.3. Protective Immune Response in Leishmania Infection

The main host defense mechanism against intracellular pathogens is macrophage activation by IFN-γ, leading to the consequent killing of infectious agents. Neutrophils, the first cell type to migrate to the *Leishmania* penetration site, are able to kill *L. braziliensis*. However, as parasites are not completely eradicated by polymorphic nuclear cells, they penetrate and survive inside macrophages. Based on CD14 and CD16 expression, monocytes are classified as classic, intermediate or pro-inflammatory, and non-classic. While *Leishmania* killing is mainly mediated by classic monocytes, intermediate monocytes are the predominant source of pro-inflammatory cytokine production [27,28]. *Leishmania* killing by macrophages is mediated mainly by nitric oxide (NO) and reactive oxygen species (ROS), e.g., superoxide anion (O^2−^), molecules produced during oxidative burst in response to phagocytosis. CL and ML patients produce high levels of IFN-γ, which synergize with TNF to induce the production of superoxide and NO, contributing to *Leishmania* control. In CL, macrophages produce ROS and NO, and macrophage activation contributes to parasite killing; nonetheless, enough *Leishmania* remain in host cells to allow infection to progress to disease.

Oxidative burst by monocytes, as determined by dihydrorhodamine (DHR) expression, is enhanced in CL monocytes compared with healthy subjects (HS) [9]. However, when DPI, an inhibitor of NADP oxidase, and L-NMMA, an iNOS inhibitor, were added to cell cultures, decreased oxidative burst and higher numbers of amastigotes in monocytes were documented exclusively in cells incubated with the NADP oxidase inhibitor [9]. While it is known that iNOS is expressed in the tissue of CL patients, in contrast to what is observed in other species of *Leishmania*, the ability of NO to kill *L. braziliensis* in human cells remains limited [9,28]. Moreover, NO expression in CL has also been associated with pathology [9]. Parasite persistence in *L. braziliensis*-infected monocytes does not occur due to the presence of superoxide dismutase, as low levels of this enzyme are observed in CL patients [23].

Macrophages play a dual role in *Leishmania* infection: while they indeed kill parasites, they also offer safe harbor for parasite survival. M1 and M2 macrophage subsets and macrophage plasticity have been well documented. M1 macrophages are pro-inflammatory and associated with infection control, whereas M2, or alternatively activated macrophages, are permissive to the growth and survival of intracellular pathogens [29]. Human M2 macrophages are mainly identified by the expression of the mannose receptor (CD206) and CD163. Although a potent Th1 immune response was induced by C57BL/6 mice infected with *L. major Seidman* (LmSd), parasites were observed to persist in CD206hi M2 macrophages [30].

Monocytes from subjects with SC *L. braziliensis* infection demonstrate a greater ability to control parasite proliferation than CL cells, thus preventing the development of disease [14]. Of note, lower amounts of IFN-γ and TNF are produced in these subjects compared to CL patients. Nevertheless, decreased parasite internalization and multiplication are observed in monocytes from SC individuals versus CL patients, indicating that monocytes from individuals with SC infection kill *Leishmania* via an innate immune response despite absent or low IFN-γ production. Future studies should attempt to comprehensively investigate how monocytes/macrophages from individuals with SC infection effectively kill *Leishmania*.

*Leishmania* killing is mediated by monocytes/macrophages following activation by IFN-γ produced by CD4^+^ T cells. In addition to CD4^+^ T cells, IFN-γ is also produced by NK and CD8^+^ T cells; in fact, CD8^+^ T cells in individuals with SC *L. braziliensis* infection were shown to express more IFN-γ than CD8^+^ T cells from CL patients. While cytotoxic molecules produced by NK cells may kill *L. aethiopica*, evidence indicating that NK or cytotoxic CD8^+^ T cells may participate in *L. braziliensis* killing is lacking. Interestingly, cytotoxic CD8^+^ T cells from CL patients were shown to kill parasite-infected cells but did not actually kill *L. braziliensis* parasites [31]. Hence, both NK and CD8^+^ T cells have been more strongly linked to pathology than protection in *L. braziliensis* infection [28,31,32,33]. IL-12 is the most important cytokine inducer of IFN-γ production by CD4^+^ T cells and NK cells. While adjuvants and proteins from *L. braziliensis* capable of inducing a Th1 immune have expectedly been identified [34,35], no vaccine for *L. braziliensis* infection has been developed.

### 2.4. Tissue Damage and Ulcer Development in Cutaneous Leishmaniasis

**Figure 2:**
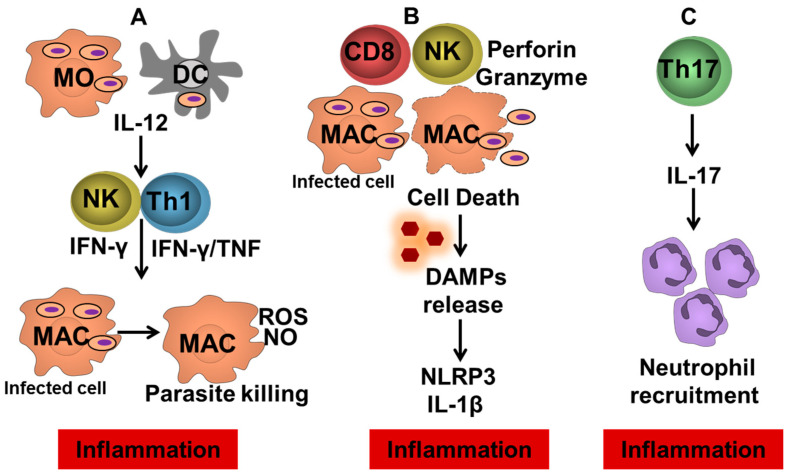
Inflammation pathways leading to ulcer development in tegumentary leishmaniasis. (**A**) Infected dendritic cells and monocytes migrate to the lymph nodes and promote the differentiation of CD4+ Th1 cells by producing IL-12. On the lesion site, Leishmania-specific CD4+ T cells and NK producing IFN-γ that are involved in parasite control by macrophage activation but are also related to chronic inflammation and lesion development. (**B**) Cytotoxic CD8+ T cells and NK cells promoting the killing of Leishmania-infected cells leading to the release of damage-associated molecular patterns (DAMPs) and consequently NLRP3 inflammasome activation and IL-1β production. (**C**) Th17 cells producing IL-17, cytokine associated with neutrophil recruitment, and enhancement of inflammatory molecules, contributing to pathology.

The main mechanisms leading to pathology in ATL are shown in Figure 2. Tissue samples of CL ulcers contain macrophages, NK cells, CD4^+^ T cells, CD8^+^ T cells and B cells. CD4^+^ T cells, as well as CD3^+^CD4^-^CD8^−^ (double negative T cells), produce high amounts of IFN-γ. A direct correlation was observed between the frequency of lymphocytes expressing IFN-γ or TNF and the lesion area in CL patients [8]. More recently a positive correlation between lesion size in CL and the frequency of a subset of senescent CD4^+^ T cells (CD45RA^+^CD27^−^) was also observed. These senescent T cells express high levels of pro-inflammatory cytokines in addition to cutaneous leucocyte-associated antigen (CLA), a skin homing receptor [36]. However, as CD4^+^ T cells are the main source of IFN-γ in *L. braziliensis* and macrophage activation by IFN-γ is a known mechanism of Leishmania killing, it cannot be ruled out that the inflammatory reaction mediated by CD4^+^ T cells predominantly participates in disease protection rather than pathology. Interestingly, around 4% of patients with CL present a negative DTH test against *Leishmania* antigens. Cells from DTH-negative CL patients were found to produce lower amounts of IFN-γ and TNF than individuals with a positive DTH reaction [37]. However, the ulcerated lesions presented by these patients are similar to those observed in patients presenting a potent Th1 immune response. Moreover, PBMCs, as well cells biopsied from DTH-negative CL patients, were observed to produce similar levels of other pro-inflammatory cytokines, as well as molecules involved in the inflammation and pathology associated with CL, including IL-1β, IL-6, granzyme B, perforin and metalloproteinase 9 (MMP-9). This finding indicates that an impaired Th1 immune response in this subgroup of patients does not attenuate pathology, suggesting that CD4^+^ T cells may not play a crucial role in the pathology of *L. braziliensis* infection.

CD8^+^ T cells exert inflammatory, cytotoxic and regulatory activities. To expand our knowledge of CD8^+^ T cell function in CL, we performed co-culturing of CD8^+^ T cells with uninfected and *L. braziliensis*-infected monocytes for 5 days. For viable parasite determination, cells were washed, the medium was replaced by Schneider’s medium, and after an additional 5 days of culturing the number of extracellular mobile promastigotes were counted. While the number of viable amastigotes was similar in CL-infected monocyte co-cultured or not with autologous CD8^+^ T cells, CD8^+^ T cells from subjects with SC infection co-cultured with infected monocytes exhibited a 50% decrease in the number of viable promastigotes compared to monocytes cultured in the absence of CD8^+^ T cells, indicating that the CD8^+^ T cells from subjects with SC infection contribute to parasite killing in infected macrophages [31]. While IFN-γ expression by CD8^+^ T cells was higher in SC subjects, the frequency of CD8^+^ T cells expressing granzyme was higher in CL patients than in SC subjects. Moreover, the frequency of *L. braziliensis*-infected monocytes expressing annexin was higher in co-cultures with CD8^+^ T cells from CL compared to co-cultures of infected monocytes with CD8^+^ T cells from SC individuals [31]. Altogether, these data indicate that CD8+CD8^+^ T cells obtained from CL patients predominantly participate in disease pathology, and do not aid in reducing parasite burden [31]. In addition to the involvement of CD8^+^ T cells in disease pathology, we documented that NK cells play an important role in the tissue damage associated with CL [32]. The frequency of NK cells is higher than CD8^+^ T cells in CL patients, and NK cells were observed to express more granzyme B and perforin than CD8^+^ T cells from CL patients. Furthermore, the frequency of degranulating NK cells, as evaluated by expression of CD107a, was higher than degranulating CD8^+^ T cells in CL lesions. Indeed, NK cells were found to exhibit 7-fold greater cytotoxicity than CD8+CD8^+^ T cells in CL [32].

Patients with E-CL, for whom diagnosis is performed prior to the appearance of skin ulceration, present a weak inflammatory reaction in comparison to patients diagnosed following the establishment of a cutaneous ulcer. The histopathological analysis demonstrated more intense inflammation associated with the recruitment of CD8^+^ T cells to the ulcer site in CL patients compared to those with E-CL. While more than 50% of CD8^+^ T cells in CL present a cytotoxic pattern expressing granzyme A in CL, only 20% of CD8^+^ T cells in E-CL were found to express this enzyme [18]. We recently showed that CD8^+^ T cells release extracellular traps, and that these cells, as well as neighboring cells, express the cytotoxic molecule CD107a. Moreover, the frequency of CD8^+^ T cells expressing extracellular traps was not only found to be higher in CL compared to E-CL, but also in ML lesions compared to CL ulcers [38]. These data support the notion that CD8^+^ T cells expressing extracellular traps participate in tissue destruction during the progression from E-CL to CL, as well as in the pathogenesis of ML.

The critical requirements underlying the participation of CD8^+^ T cells in lesion development in CL were shown in RAG knock-out (KO) mice lacking both B and T cells. In comparison to wild-type mice, *L. braziliensis*-infected RAG KO mice develop pathology slowly, manifesting minimal lesions despite high parasite burden [33]. Moreover, while the reconstitution of CD4^+^ T cells in RAG KO mice was found to be protective, mice receiving CD8^+^ T cells then developed severe lesions [33]. By applying genome-wide transcriptional profiling in tissue obtained from CL ulcers, we identified an elevated transcription of granzyme A, granzyme B, perforin, NLRP3 and IL-1β compared to healthy skin samples. Moreover, IL-1β expression was shown to directly correlate with levels of granzyme A, granzyme B and perforin. The NLRP3 inflammasome is an intracellular protein complex that promotes caspase 1 activation and the release of active IL-1β. Recently, we showed that CD8^+^ T cells induce immunopathology by NLRP3 inflammasome activation and IL-1β production in *L. braziliensis* infected mice, and both genetic and pharmacological inhibitors of NLRP3 reduce pathology. Indeed, cytotoxic CD8^+^ T cells promote killing of *Leishmania*-infected cells leading to the release of damage-associated molecular patterns (DAMPs), and consequently NLRP3 inflammasome activation and IL-1β production [39]. Glyburide, an ATP-sensitive K+ channel inhibitor used in the treatment of type 2 diabetes, is capable of inhibiting the NLRP3 inflammasome and decreasing IL-1β production. The addition of glyburide to PBMC cultures from CL patients stimulated with SLA or to biopsies from CL ulcer cultures did not modify levels of IFN-γ, IL-6 or IL-10, yet decreased the production of IL-1β, IL-17 and TNF [40].

Granzyme B, a serine protease secreted by cells stimulated with IL-2, IL-15 and IL-21, is responsible for cytotoxic-granule-mediated cell death pathway activation. As IL-15 promotes granzyme B-dependent CD8^+^ T cell cytotoxicity, tofacitinib, a JAK inhibitor that reduces IL-15 synthesis, was evaluated with respect to its ability to attenuate pathology in *L. braziliensis*-infected RAG mice reconstituted with CD8^+^ T cells. Both topical and systemic administration of tofacitinib was shown to protect mice from developing severe CL lesions [41].

### 2.5. Downmodulation of the Inflammatory Response in the Treatment of Tegumentary Leishmaniasis

The treatment of ATL has mainly entailed the use of meglumine antimoniate at a dose of 20 mg/Kg/weight/day for 20 days. Patients who present failure to therapy receive a second course. In case of failure to the second course, treatment with amphotericin B is recommended. It was recently demonstrated that orally administrated miltefosine is effective in the treatment of CL caused by *L. braziliensis* [42]. The use of meglumine antimoniate implies important limitations. In some endemic areas, such as Corte de Pedra, failure has been documented in up to 60% of CL patients. In those who achieve cure, healing time is prolonged, ranging from 60 to 90 days. Moreover, antimony is administered by the intravenous route and has been associated with relevant adverse reactions. While cure is achieved by miltefosine in up to 76.6% of CL patients, patients suffer teratogenic effects and high cost, in addition to nausea and vomiting [42]. Although highly effective, Amphotericin B requires hospital admission for drug administration and may provoke adverse reactions, e.g., kidney injury, thereby limiting its use, especially in poorer areas where ATL is endemic. As ulcer development in ATL is provoked by an exaggerated host inflammatory response, ATL therapy may be improved through the use of drugs capable of downmodulating the immune response, in combination with chemotherapy to kill parasites. The potential candidates include granulocyte macrophage colony stimulating factor (GM-CSF), the blockade of IL-1β receptor using monoclonal antibodies, glyburide, tofacitinib and candesartan.

In a randomized controlled clinical trial, subcutaneous or topical GM-CSF associated with meglumine antimoniate was shown to be more effective than antimony alone in the treatment of CL, resulting in decreased healing time. While a 50% cure rate was achieved in patients who received meglumine antimoniate alone, combined therapy with meglumine antimoniate plus topical GM-CSF achieved 100% healing [43]. Combined therapy is also found to be effective in the treatment of DL. However, as the granulocyte colony stimulating factor more effectively enhances neutrophil production than GM-CSF, GM-CSF is no longer commercially available. Pentoxifylline is a vasodilator that decreases TNF synthesis; when used in association with meglumine antimoniate, it was shown to be more effective than antimony alone in the treatment of ML, and is now recommended by Brazilian health authorities for ML treatment. Sm29 is a protein expressed in the membrane of *Schistosoma mansoni*, which downmodulates the inflammatory response in CL patients. In an ongoing randomized controlled clinical trial, partial results showed a cure rate of 43% using meglumine antimoniate to treat CL, while therapy involving topical Sm29 encapsulated in gold nanoparticles plus meglumine antimoniate cured 83% of the enrolled patients with CL. Moreover, while the mean healing time in patients receiving antimony was 115 (59–177) days, those who received Sm29 plus antimony achieved cure in 47 (33–90) days.

Toll-like receptors (TLR) recognize pathogen-associated molecular patterns (PAMPs) expressed by infectious agents. TLR signaling leads to the transcription of inflammatory mediators, such as IL-1β, TNF and chemokines. While TLRs play a significant role in host defense mechanisms, the neutralization of TLRs can also attenuate pathology in diseases associated with chronic inflammation. As our previous work has demonstrated, the neutralization of TLR-2 and TLR-4 in monocytes from CL patients decreases *L. braziliensis* internalization and the production of pro-inflammatory cytokines [44]. Therefore, we suggest that TLR-2 and TLR-4 antagonists, such as candesartan, should be evaluated with respect to a potential role as adjuvants in CL therapy.

Other potential drugs that may be considered in combination with chemotherapy to treat CL are glyburide and tofacitinib [40,41]. Glyburide, which blocks NRLP3 inflammasome activation, is an antidiabetic drug approved by the FDA for the treatment of type 2 diabetes. Tofacitinib, an IL-15 inhibitor, decreases granzyme B production. Both glyburide and tofacitinib have been shown to attenuate CL pathology in mice infected with *L. braziliensis* and warrant evaluation in combination with chemotherapy in the treatment of human CL. 

## 3. Concluding Remarks

The main limitation in controlling *L. braziliensis* infection is the parasite’s ability to survive inside infected cells, despite the presence of *Leishmania*-killing mechanisms known to be effective. The infected macrophages produce ROS and NO and express TLRs and inflammatory cytokines, yet possess a limited ability to kill *L. braziliensis*. Parasite persistence enhances the activity of macrophages, as well as CD4^+^ and CD8^+^ T cells and NK cells; however, instead of providing protection, pathologic subsets of these cells are generated. CD8^+^ and NK cell subsets with a cytolytic profile kill *Leishmania*-infected cells via granzyme and perforin, leading to the release of DAMPs, followed by NLRP3 inflammasome activation and IL-1β production. Together, these circumstances induce tissue pathology and exacerbate ulcer development.

Meglumine antimoniate is considered the first-line drug for ATL treatment in Latin America; however, increasing reports of therapeutic failure and prolonged ulcer healing time represent relevant limitations. As exaggerated inflammation is the underlying cause of pathology, drugs that downmodulate the inflammatory reaction, used in combination with antimony, have shown demonstrable success in animal models and humans. This strategy to humans is a worthy pursuit potentially capable of increasing cure rates and decreasing the healing time associated with ATL.

## Figures and Tables

**Table 1:** Cytokine profiles in different clinical forms of *L. braziliensis* infection.

Cytokines	CL	ML	Atypical CL	DL	SC
IFN-γ	1047 ± 358	1456 ± 437	731 ± 463	438 ± 178	237 ± 104 *
IL-4	157 ± 181	201 ± 165	108 ± 124	115 ± 163	98 ± 77
TNF	983 ± 252	1369 ± 376	504 ± 192	318 ± 204	119 ± 69 *
IL-17	187 ± 139	329 ± 105	587 ± 266	98 ± 107	81 ± 115 **

Peripheral blood mononuclear cells from patients with different clinical forms of *L. braziliensis* infection were stimulated with soluble Leishmania antigen (5 µg/mL) for 48 h, 37 °C, 5% CO_2_. Cytokines were determined in culture supernatants by ELISA. * *p* < 0.05 CL and ML versus DL and SC, ** *p* < 0.01 Atypical CL versus CL, DL and SC.

## Data Availability

Not applicable.
